# Dynamic Allocation of C-V2X Communication Resources Based on Graph Attention Network and Deep Reinforcement Learning

**DOI:** 10.3390/s25165209

**Published:** 2025-08-21

**Authors:** Zhijuan Li, Guohong Li, Zhuofei Wu, Wei Zhang, Alessandro Bazzi

**Affiliations:** 1 School of Computer and Big Data, Heilongjiang University, Harbin 150080, China; lizhijuan@hrbeu.edu.com (Z.L.); 2231997@s.hlju.edu.cn (G.L.); zhangwei_jsj@hlju.edu.cn (W.Z.); 2 Postdoctoral Program of Heilongjiang Hengxun Technology Co., Ltd., Xuefu Road, Harbin 150090, China; 3 Shandong Hengxun Technology Co., Ltd., Miaoling Road, Qingdao 266100, China; 4 Department of Computer Science and Technology, Harbin Engineering University, Harbin 150001, China; 5 WiLab, CNIT/DEI, University of Bologna, 40136 Bologna, Italy; alessandro.bazzi@unibo.it

**Keywords:** dynamic vehicular networks, vehicle-to-vehicle, graph attention networks, reinforcement learning, advantage actor–critic

## Abstract

Vehicle-to-vehicle (V2V) and vehicle-to-network (V2N) communications are two key components of intelligent transport systems (ITSs) that can share spectrum resources through in-band overlay. V2V communication primarily supports traffic safety, whereas V2N primarily focuses on infotainment and information exchange. Achieving reliable V2V transmission alongside high-rate V2N services in resource-constrained, dynamically changing traffic environments poses a significant challenge for resource allocation. To address this, we propose a novel reinforcement learning (RL) framework, termed Graph Attention Network (GAT)-Advantage Actor–Critic (GAT-A2C). In this framework, we construct a graph based on V2V links and their potential interference relationships. Each V2V link is represented as a node, and edges connect nodes that may interfere. The GAT captures key interference patterns among neighboring vehicles while accounting for real-time mobility and channel variations. The features generated by the GAT, combined with individual link characteristics, form the environment state, which is then processed by the RL agent to jointly optimize the resource blocks allocation and the transmission power for both V2V and V2N communications. Simulation results demonstrate that the proposed method substantially improves V2N rates and V2V communication success ratios under various vehicle densities. Furthermore, the approach exhibits strong scalability, making it a promising solution for future large-scale intelligent vehicular networks operating in dynamic traffic scenarios.

## 1. Introduction

### 1.1. Motivation

In the era of smart cities, intelligent transport systems (ITSs) enhance traffic efficiency, alleviate vehicular congestion, and improve road safety, with vehicle-to-everything (V2X) communication—encompassing vehicle-to-vehicle (V2V), vehicle-to-infrastructure (V2I), vehicle-to-network (V2N), and vehicle-to-pedestrian (V2P)—serving as a key enabler [1,2,3]. V2V communication is essential for direct, ultra-low-latency data exchange between vehicles, enabling safety-critical functions such as collision avoidance, whereas V2N supports high-throughput interactions with cellular infrastructure or base station (BS) for applications including real-time traffic updates and remote diagnostics. However, interlink interference and limited spectrum resources make it challenging to balance high V2N data rates with robust V2V reliability. Effective resource block (RB) allocation can mitigate interference and latency, optimizing the trade-off to achieve efficient utilization of the spectrum, high V2N rates, and reliable V2V connections, thereby enhancing performance in dense and dynamic vehicular networks.

In dynamic vehicular environments, traditional radio resource management (RRM) methods struggle to perform effectively due to high mobility, rapidly changing network topologies, and challenging wireless channel conditions. Centralized strategies, such as LTE-V2X Mode 3 and new radio-V2X (NR-V2X) Mode 1, face scalability limitations caused by heavy signaling overhead and reliance on precise global channel state information (CSI) [4]. In contrast, distributed strategies, including LTE-V2X Mode 4 and NR-V2X Mode 2, are hindered by limited local observations and the absence of centralized coordination, leading to frequent collisions and unreliable communication links [5].

The application of reinforcement learning (RL) to resource management in vehicular networks has recently attracted considerable attention. RL-based frameworks leverage agent-environment interactions to learn optimal allocation policies [6,7], enabling agents to adapt to highly dynamic scenarios and optimize long-term objectives without requiring explicit models [8]. However, traditional RL methods, especially those based on independent learning agents with only local observations, are constrained by partial observability, nonstationary environments, and insufficient modeling of inter-agent interactions.

In dynamically changing traffic environments, complex resource competition and channel interference exist among V2V communication links. Ignoring the relational structure among vehicles leads to suboptimal decision-making, making single-agent RL insufficient to address these challenges [9]. Each agent (i.e., a V2V link) must consider not only its local state information—such as channel gain, remaining transmission time, and resource demands—but also, more critically, the potential interference from neighboring links sharing the same spectrum resources. When agents rely solely on local information for policy learning, resource conflicts often arise in high-density dynamic environments, substantially reducing communication success ratios and overall system throughput.

Traditional RL methods [10], such as double Q-learning (DQN) or basic Actor–Critic strategies, typically model states as individual observations, lacking the ability to incorporate adjacency relationships in communication topologies or capture interference structures between links. Policy networks constructed solely from traditional feature inputs, therefore, struggle to capture critical interference information embedded in the network structure, leaving learned policies prone to local optima and self-interested behavior.

Vehicular networks inherently exhibit graph-structured characteristics: interference relationships among links naturally form a graph, with edges representing resource conflict probabilities or interference relationships. The primary objective of this paper is to coordinate V2V and V2N communications to ensure their coexistence while maximizing V2V reliability and meeting latency requirements, and simultaneously minimizing V2V interference to V2N links. We quantify V2V reliability using the success reception ratio.

To this end, we introduce the Graph Attention Network (GAT) as an interference-aware module in state processing [11]. The GAT models the communication environment as a graph in which each node corresponds to a V2V link and edges denote potential interference. Its core mechanism employs attention weights to assess the importance of each neighboring link for the current link’s policy decision, aggregating neighbor features with weighted contributions to generate a state representation enriched with structural information. This representation incorporates both local state information and structural context—specifically, whether neighboring links conflict and the significance of such conflicts—enabling the policy network to achieve more effective global coordination.

Furthermore, we adopt a dynamic graph construction mechanism. At each time step, the adjacency matrix is reconstructed based on current vehicle positions, channel states, and resource usage, producing a communication graph that reflects the real-time interference landscape. Compared to static adjacency graphs, dynamic construction more accurately captures evolving conflict risks among links, ensuring that the information aggregated by the GAT remains up to date. This approach substantially enhances the generalization capability and environmental adaptability of the policy network.

### 1.2. Related Work

Early works, such as [8,12,13,14] introduced Q-Learning and DQN methods for distributed resource allocation in V2V networks. These approaches enabled each vehicle or communication link to independently learn resource selection policies, optimizing throughput or reliability while minimizing interference. However, they often assumed stationary environments or negligible inter-agent action impacts, which do not hold in dense vehicular networks where mutual interference is prevalent. To tackle issues arising from nonstationary and partial observability, some studies explored RL methods, including independent Q-Learning [12], Double Q-Learning [15], and Deep Deterministic Policy Gradient (DDPG) [14]. More advanced architectures, such as Multi-Agent DDPG (MADDPG) [16], explicitly modeled interactions among multiple agents to improve coordination and overall system performance in V2X. Despite progress, existing RL-based resource allocation methods often treat agents as isolated learners or assume limited interaction modeling, constraining their ability to dynamically predict and mitigate interference. Additionally, traditional RL frameworks typically assume a fixed number of agents and static state-action spaces, making them ill-suited for dynamic vehicle densities and fluctuating network topologies.

Given the limitations of traditional RL in modeling inter-agent dependencies, researchers have increasingly turned to Graph Neural Networks (GNNs) to capture the complex relational structures within vehicular networks [17,18]. GNNs, including Graph Convolutional Networks (GCNs) [19] and GraphSAGE [20], provide powerful tools for learning representations on graph-structured data, where nodes represent vehicles or links, and edges denote interference, proximity, or communication quality. In vehicular network applications, GNNs have been used for link prediction [21], channel allocation, and resource allocation [22,23,24]. By aggregating information from neighboring nodes, GNNs enables vehicles to construct richer, context-aware representations of their local environment, leading to more informed and coordinated decisions. However, most existing GNN-based methods assume static or slowly evolving graph topologies. In dynamic vehicular networks, where high mobility, varying vehicle numbers, and rapidly changing relationships prevail, maintaining and updating graph structures pose significant challenges. Moreover, many GNN methods are integrated into centralized frameworks, where a central controller aggregates global information for decision-making. While effective in some scenarios, centralized architectures face scalability and latency issues, making them unsuitable for fully distributed V2V communication systems.

Recent efforts have explored dynamic graph learning, proposing techniques like dynamic graph convolutional networks [11] and attention-based dynamic edge updates [12] to efficiently capture and update evolving neighbor relationships in vehicular communication, reflecting changing interference patterns and mobility behaviors. Nevertheless, integrating dynamic graph learning into RL for distributed vehicular networks remains relatively unexplored. Most existing GNN-RL hybrid methods either fix graph structures during training or update them infrequently, limiting their responsiveness to real-time environmental changes. Thus, there is an urgent need for frameworks that can (1) dynamically adapt to varying vehicle densities and network topologies, (2) effectively model and leverage inter-agent relationships through graph-based learning.

### 1.3. Contributions

The main contributions of this work are summarized as follows:

- Firstly, we adopt a GAT to extract global features, shifting the optimization objective from individual vehicle performance to system-wide optimality across the entire vehicular network.
- Secondly, we dynamically update neighbor relationships based on real-time vehicle positions to accurately capture current interference patterns between vehicles.
- Thirdly, we propose a novel GAT-Advantage Actor–Critic (GAT-A2C) RL framework, pioneering the integration of GAT with the Advantage Actor-Critic (A2C) algorithm. This architecture dynamically adapts to positional changes, communication states, and interference fluctuations among neighboring vehicles, enabling optimized resource allocation for both V2V and V2N links.
- Lastly, we conduct extensive experimental evaluations across diverse vehicular scenarios with varying densities. Results demonstrate that our GAT-A2C framework outperforms existing methods in key metrics (including V2N rate and V2V success ratio), particularly excelling in high-density environments. The solution further exhibits robust adaptability and superior scalability across all tested vehicle densities.

### 1.4. Organization

The remainder of this paper is organized as follows: In Section 2, we present a detailed system model for the communication scenario and interference computation. Section 3 introduces the design of the GAT. In Section 4, we detail the methodology of GAT-A2C, the training process of A2C, and system implementation considerations. Section 5 presents experimental settings and experimental results. Finally, Section 6 concludes the paper.

## 2. System Model, Problem Formulation, and Solution Scheme

### 2.1. System Model

**Figure 1:**
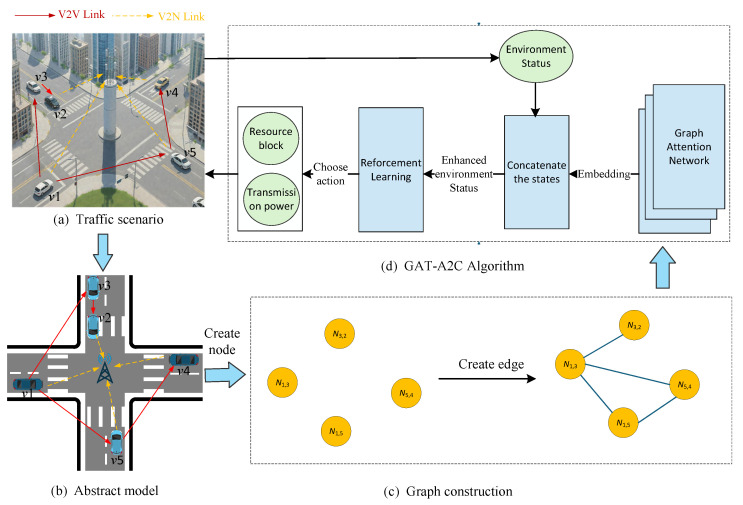
System model and overall solution scheme. (**a**) Traffic scenario at intersections (vehicles are randomly distributed on the roads and a BS is located at the center of the scenario); (**b**) The abstract model, (**c**) Graph construction (a subset of V2V links forms the nodes, and edges represent interference relationships between these links); (**d**) GAT-A2C Algorithm.

This paper focuses on resource selection for V2V and V2N communications in an intersection scenario, as illustrated in Figure 1a. The V2N uplink and V2V communications share the orthogonal frequency-division multiplexing (OFDM) band in an in-band overlay manner [20]. In the communication system, the received signal power is directly proportional to the transmit power while being modulated by channel gain, which consists of three components: path loss, shadowing, and fast fading. The power of the received signal can be expressed as Equation (1):

(1) $$P_{\mathrm{Tx},\mathrm{Rx}}=P_{\mathrm{Tx}}\cdot G_{\mathrm{Tx}}\cdot G_{\mathrm{Rx}}\cdot G_{\mathrm{Ch}},$$

where $P_{\mathrm{Tx}}$ is the transmit power, $G_{\mathrm{Tx}}$ and $G_{\mathrm{Rx}}$ denote the antenna gains of the transmitter and receiver, respectively, and $G_{\mathrm{Ch}}$ is the channel gain, which can be expressed as Equation (2):

(2) $$G_{\mathrm{Ch}}=10^{-\frac{PL+L_{s}+L_{f}}{10}},$$

where PL, $L_{s}$, and $L_{f}$ represent path loss, shadowing, and fast fading, respectively.

Assuming a scenario with *m* V2N links, denoted by $L^{V2N}=\left\{L_{1}^{V2N},L_{2}^{V2N},\ldots ,L_{m}^{V2N}\right\}$, *n* connected vehicles, denoted by $V=\{V_{1},V_{2},\ldots ,V_{n}\}$, and *k* pairs of V2V links, denoted by $L^{V2V}=\left\{L_{1}^{V2V},L_{2}^{V2V},\ldots ,L_{k}^{V2V}\right\}$. For a V2N or V2V link whose receiver is vehicle $V_{i}$, the signal-to-interference-plus-noise ratio (SINR) is calculated in Equation (3):

(3) $${\mathrm{SIN}R}_{V_{i}}=\frac{P_{V_{i}}}{\sum_{j\neq i}\delta_{j,i}I_{V_{j}}+\delta_{c,i}I_{c}+N_{0}}$$

where $P_{V_{i}}$ denotes the signal power received from the tagged link; $I_{V_{j}}$ and $I_{c}$ represent the interference power from vehicle $V_{j}$ and BS, respectively; $\delta_{j,i}$ and $\delta_{c,i}$ denote indicator variables, taking the value 1 when their corresponding resource blocks overlap with those of the tagged link, and 0 otherwise; $N_{0}$ represents the noise power.

Thus, according to Shannon’s second theorem, the channel capacity of the tagged link is given by Equation (4):

(4) $$C=B\log_{2}\left(1+{\mathrm{SINR}}_{V_{i}}\right)$$

where *B* denotes the channel bandwidth.

In cellular-V2X (C-V2X) communication systems, the transmission success ratio for V2V communication is a key metric for assessing reliability. It represents the proportion of successfully transmitted packets to the total number of packets sent, reflecting the communication performance of V2V links in specific scenarios. It is defined as the ratio of the number of packets correctly received by the receiver to the total number of packets sent by the transmitter within a given time period. It is a critical indicator of reliability in V2V communication, particularly in complex scenarios (e.g., intersections) with interference, channel fading, and resource competition. Suppose vehicle $V_{i}$ sends packets to vehicle $V_{j}$ via a V2V link. Let $N_{\mathrm{sent}}$ be the total number of packets sent by the transmitter $V_{i}$; $N_{\mathrm{received}}$ be the number of packets correctly received by the receiver $V_{j}$. The transmission success ratio for V2V communication is defined as Equation (5):

(5) $$R_{V2V}=\frac{N_{\mathrm{received}}}{N_{\mathrm{sent}}}$$

where $R_{V2V}\in \left[0,1\right]$, and a value closer to 1 indicates higher communication reliability. The $R_{V2V}$ is modeled as $R_{V2V}=\frac{1}{1+e^{-k(\mathrm{SIN}R-\gamma_{\min})}}$, where $\gamma_{\min}$ is the minimum SINR threshold for decoding, and *k* adjusts sensitivity. The larger the SINR, the higher the probability of transmission success.

### 2.2. Problem Formulation

As shown in Equation (3), SINR, which determines the probability of successful packet decoding, is defined as the ratio of received signal power, governed by transmit power and channel gain, to the sum of interference and noise. Resource block selection and transmission power control significantly impact the SINR. Resource block selection optimizes spectrum allocation to enhance throughput and spectrum efficiency while reducing channel congestion and co-channel interference through orthogonal assignments and exclusivity constraints, ensuring communication reliability. Transmission power control balances the trade-off between improving received signal quality and minimizing interference to other links, as excessive power, while enhancing signal strength, exacerbates interference, thereby degrading overall system performance. The transmission success ratio ($R_{V2V}$) and channel capacity are closely tied to the SINR. Optimized resource block allocation and power control significantly enhance system performance, thereby boosting the reliability of V2V and V2N communications. Then, the objective of the study is to optimize resource block selection and transmission power selection to maximize system efficiency while minimizing channel congestion and resource conflicts in C-V2X networks.

### 2.3. Overall Solution Scheme

We designed the GAT-A2C framework to address the resource allocation problem. In the framework, a hybrid reinforcement learning and graph neural network approach is employed for V2V resource allocation in vehicular networks, as shown in Figure 1d. The framework integrates GAT with A2C reinforcement learning to optimize power control and resource block allocation for V2V communications. Graph construction model, GAT module, RL agent are the core components. In graph construction model, a graph-based representation of the vehicular network is constructed, as shown in Figure 1c. The graph construction process dynamically updates based on vehicle positions and communication demands, which is detailed in Section 3.1. The GAT processes the vehicular network graph to generate node embeddings that capture the spatial relationships and interference patterns. The GAT employs multi-head attention mechanisms to aggregate information from neighboring nodes, producing 20-dimensional feature representations that enhance the reinforcement learning state space. Finally, an Actor–Critic agent implements the decision-making process for resource allocation. The agent receives enhanced state information combining traditional channel states with GAT-generated embeddings, and outputs actions for power level selection and resource block assignment.

## 3. Design of Graph Attention Network

In traffic scenarios, V2V links encounter non-uniform interference patterns, with some links being subject to strong interference while others are affected only weakly. We leverage the attention mechanism of GAT to adaptively learn which links contribute more or less interference. This allows the vehicle to effectively avoid heavily interfering neighbors during action selection, thereby reducing link-level interference and improving overall communication performance. In this section, we introduce graph construction and GAT model.

### 3.1. Graph Construction

#### 3.1.1. Principle of Graph Construction

In this paper, we model the vehicular network as a graph. Each node in the graph, denoted as $N_{i,j}$, represents a unidirectional V2V communication link from transmitter *i* to receiver *j*. The edge, denoted as $E_{x,y}$, represents the interference exerted from node *x* to node *y*. A complete graph is unsuitable in this context, as vehicles in a vehicular network often transmit messages in a broadcast manner, causing the number of nodes to grow quadratically with the number of vehicles, which in turn leads to significant computational overhead during subsequent processing.

To address the scalable problem of the complete graph, Ji et al. [20] constructed a graph based on the communication relationship between vehicles, and their simulation results demonstrated its effectiveness. Thus, this paper adopts the same approach to model the vehicular network. For each vehicle, three output links are selected whose destination vehicles belong to the nearest 20% subset of all vehicles. This approach reduces the number of nodes to a linear scale, approximately three times the number of vehicles. Then the graph structure can be expressed as Equation (6):

(6) G=(N,E)

where N is the set of nodes that represents the unidirectional V2V links. E represents the set of all edges in the graph. An edge is established between the corresponding nodes if mutual interference exists between two links.

#### 3.1.2. Graph Node State

The feature vector initialized for each node *N* is denoted in Equation (7):

(7) $$S_{N}=\left[G_{\mathrm{Ch}}^{V2N},G_{\mathrm{Ch}}^{V2V},I_{N}\right],$$

where $G_{\mathrm{Ch}}^{V2N}$ and $G_{\mathrm{Ch}}^{V2V}$ represent the channel gain of the V2N and V2V communications, respectively; $I_{N}$ denotes the total interference power experienced by the node *N*.

#### 3.1.3. Dynamic Adjacency Matrix for Graph Expression

According to the principle of graph construction, we adopt a link-level adjacency matrix *A* with the size of $|L^{V2V}\left|\times \right|L^{V2V}|$ (which is 3k×3k, since three output links are sampled for each vehicle), representing the connectivity relationships between nodes of the graph. The elements of the link-level adjacency matrix *A* are defined in Equation (8):

(8) $$A_{x,y}=\left\{\begin{array}{cc} 1, & \mathrm{if}\;\mathrm{nodes}\;x\;\mathrm{and}\;y\;\mathrm{are}\;\mathrm{connected}\\ 0, & \mathrm{otherwise} \end{array}\right.$$

where $A_{x,x}\equiv 1$ to preserve the influence of the node’s own features during the attention scoring computation in GAT.

Due to the continuous motion of vehicles, neighbor relationships are not static. Thus, the link-level adjacency matrix *A* is reconstructed every *T* seconds. Meanwhile, the matrix is also updated immediately upon detecting events such as vehicle acceleration changes exceeding a threshold or significant position jumps. In order to balance communication latency and computational overhead, the update time is typically set between 0.1 s and 1 s.

### 3.2. GAT Model

After constructing the graph, we introduce the GAT model. In communication resource allocation problems, we employ GAT as an auxiliary learning module for reinforcement learning, utilizing supervised learning to capture spatial relationships and interference patterns among vehicles. The objective of GAT is to learn a mapping function defined in Equation (9)

(9) $$f:G\to R^{|N|\times d}$$

where G=(N,E) is the vehicular communication network graph defined in Equation (6), $R^{|N|\times d}$ denotes node embedding space, N represents the number of nodes and *d* is the embedding dimension. The embedding dimension *d* is the number of resource blocks in the system.

**Figure 2:**
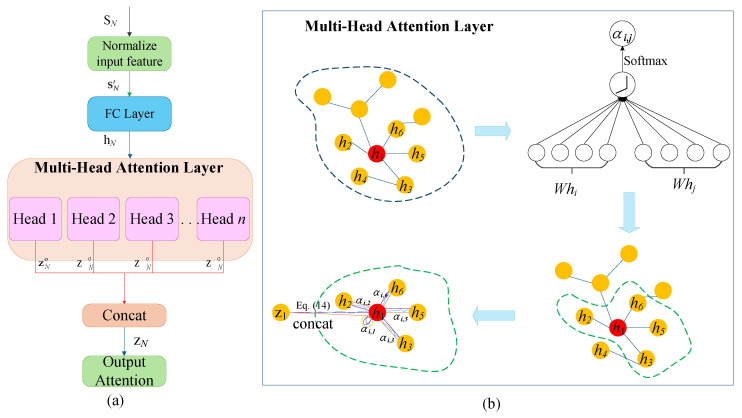
(**a**) Overall aggregation process of the GAT model employed by our model. (**b**) An illustration of multi-head attention (with K = 3 heads) by node 1 on its neighborhood.

The aggregation process in GAT describes how each node gathers information from its neighbors, computing a weighted sum, and updates its features, as illustrated in Figure 2. Specifically, the operation of each node involves the following steps:

- performing linear transformation on the features of itself and all neighboring nodes;
- calculating the attention score of itself and each neighbor;
- normalizing these attention scores as weights using softmax;
- The weights are applied to aggregate neighbor features and obtain the updated node features.

#### 3.2.1. Normalization and Linear Transformation on the Features

In this paper, the initialization of V2V link features serves as the starting point for the input of the entire system. These features are directly fed into the GAT encoding module for neighbor relationship modeling and feature aggregation, while also serving as critical inputs for subsequent Actor action generation and Critic value evaluation. Insufficient input feature information can lead to incomplete node feature extraction by GAT, thereby impacting the accuracy of resource allocation decisions and overall system performance. As previously discussed, we modeled the state of a graph node *N* as $S_{N}$. However, during the training and iteration of the GAT network, uneven feature dimensions may lead to gradient explosion issues. To accelerate training convergence and reduce the risk of gradient explosion from inconsistent feature dimensions, Equation (10) is used to normalize the initial input features of the graph nodes:

(10) $$S_{N}^{\prime }=\frac{S_{N}-\mu }{\sigma }$$

where μ and σ represent the mean and standard deviation of each feature in the training set, respectively. After normalization, feature values are centered around 0, which enhances the stability and convergence speed of GAT encoding and Actor–Critic training [25].

A linear transformation is then applied to the initial node features. The transformation operation is performed by a fully connected (FC) layer, as shown in Figure 2. This transformation maps all node features into a unified feature space, ensuring consistent inputs for the subsequent attention scoring process. The transformed feature vector for each node can be computed with Equation (11):

(11) $$h_{N}=WS_{N}^{\prime }$$

where $W\in R^{F^{\prime }\times F}$ is a shared learnable weight matrix, *F* is the original feature dimension, and $F^{\prime }$ is the dimension of $h_{N}$ after transformation.

#### 3.2.2. Attention Score Computation and Aggregation Result of a Single Attention Head

To capture the local similarity or interference strength between a node and its neighbors, GAT computes an attention score for each pair of adjacent nodes (x,y) (where $A_{x,y}=1)$ based on their feature vector with Equation (12):

(12) $$e_{x,y}=\mathrm{LeakyReLU}\left(a^{T}[h_{x}\left|\right|h_{y}]\right)$$

where $a\in R^{2F^{\prime }\times 1}$ is a learnable attention vector, || denotes vector concatenation, and LeakyReLU is the activation function. Then, to ensure that attention scores are comparable across nodes with different numbers of neighbors, the scores are normalized using the Softmax function, as shown in Equation (13):

(13) $$\alpha_{x,y}=\mathrm{Softmax}\left(e_{x,y}\right)=\frac{\exp(e_{x,y})}{\sum_{k\in N_{x,\mathrm{nbr}}}\exp\left(e_{x,k}\right)}$$

where $N_{x,\mathrm{nbr}}$ represents the set of neighbors of node *x*. The sum of the normalized attention score for all neighbors of a node equals 1, forming a probability distribution to prevent the features of any neighbor from dominating.

GAT computes an attention score $e_{x,y}$ by feeding the concatenated state features of each neighbor node pair (x,y) into a shared feedforward network, followed by normalization via Softmax to obtain the weight $\alpha_{x,y}$. This weight reflects the importance of a neighbor node to the current node’s policy generation. In V2V scenarios, a neighbor’s importance can be interpreted as its potential interference capability, with higher weights assigned to neighbors more likely to cause resource conflicts. Through this aggregation mechanism, GAT enables each link to identify neighbors posing genuine interference threats, allowing proactive avoidance of these conflict sources in subsequent policy selections.

A weighted sum of the neighboring node features is computed using the normalized attention coefficients, as given in Equation (14).

(14) $$z_{x}^{o}=\mathrm{ReLU}\left(\sum_{y\in N_{x,\mathrm{nbr}}}\alpha_{x,y}h_{y}\right)$$

where ReLU is a nonlinear activation function, and $z_{x}^{o}$ represents the context-enhanced feature representation of node *x*. By dynamically integrating information from the most important neighbors, this produces node features enriched with contextual and interference-aware information, providing a more accurate basis for subsequent action selection.

#### 3.2.3. Multi-Head Attention Mechanism

The multi-head attention mechanism enables understanding neighbor features from multiple perspectives. A multi-head attention mechanism is employed to further enhance expressiveness and stability. While using *K* attention heads, $W^{k}$ and $a^{k}$ represent the independent weight matrices and attention vectors for *k*-th head. As shown in Figure 2, each head independently computes attention coefficients and weighted aggregation outputs; intermediate layer outputs are concatenated. The formulation is presented in Equation (15):

(15) $$z_{x}=\mathrm{Concat}{\left(\mathrm{ReLU}\left(\sum_{y\in N_{x,\mathrm{nbr}}}\alpha_{x,y}^{k}h_{y}^{k}\right)\right)}_{k=1}^{K}$$

where Concat (·) denotes the concatenation of the output vectors from all *K* attention heads along the feature dimension.

### 3.3. Loss Function of GAT

We train GAT based on supervised learning. The training objective of GAT is to minimize the weighted loss function, as given in Equation (16):

(16) $$\theta^{*}=\arg\min_{\theta }L_{\mathrm{GAT}}\left(\theta \right)$$

where θ represents the parameter set of the GAT model. The loss function adopts a mean squared error (MSE), which is given in Equation (17):

(17) $$L_{\mathrm{GAT}}=\frac{1}{N}\sum_{N\in N}{\left(y_{N}-z_{N}\right)}^{2}$$

where $z_{N}$ is the output of GAT, $y_{N}$ is the label of V2V link node *N*, which reflects the comprehensive quality of the communication link, providing effective supervision for GAT. When a link actually performs an action on a certain resource block, the value returned by the environment is stored as the label of V2V link node *N*.

## 4. The GAT-A2C Model for Resource Allocation Problems

In this model, the node embeddings output by the GAT are incorporated as structured features into the state input of the RL agent, greatly enhancing the expressiveness of the state representation. In this way, the RL main network can perceive not only the communication quality, interference level, and resource demand of each link, but also its complex dependencies with neighboring nodes. This enables the agent to make more optimal resource allocation decisions. The integration of GAT and RL in this manner significantly improves the overall performance and generalization capability of the system. This section introduces the GAT-A2C model for optimizing C-V2X resource allocation, including the design of key elements in RL and A2C, as well as the overall framework of the GAT-A2C model.

### 4.1. The Design of Key Elements in RL and A2C

RL is commonly represented as a Markov Decision Process (MDP), where an agent repeatedly engages with its environment. At each time step, the agent observes the environment’s current state, $S_{t}$, and chooses an action, $a_{t}$, based on that state. In response, the environment delivers a reward, $r_{t}$, and moves to a new state, $S_{t+1}$. This sequence forms a full RL interaction cycle.

In this study, the input state of the RL agent is defined as the combination of features from the GAT and features provided by the environment. The agent responds by selecting both resource blocks and a transmission power level. The environment then returns a reward based on the V2V link interruption probability, V2N communication rate, and transmission time. This reward guides the agent to continuously optimize its action selection to maximize long-term reward, ultimately aiming to learn an optimal dynamic resource allocation strategy.

#### 4.1.1. State Space

The state feature vector of each V2V link, as obtained from the environment, is denoted in Equation (18):

(18) $$s_{t}=\left[C_{t},I_{t},G_{t},N_{t},T_{t},L_{t}\right]$$

where $C_{t}$ represents the channel capacity of the V2N link, $I_{t}$ denotes the total interference power received by the V2V link, $G_{t}$ indicates the channel gain of the local V2V link, $N_{t}$ reflects the resource occupancy status of neighboring links, $T_{t}$ stands for the remaining transmission time, $L_{t}$ represents the remaining data to be transmitted.

In addition to the state from the environment, we also incorporate the feature $z_{t}$ generated by the GAT as part of the state. In this way, the state of the agent is expressed in Equation (19):

(19) $$S_{t}=s_{t}\|z_{t}$$

where the symbol “‖” denotes concatenation.

#### 4.1.2. Action Space

Based on the collected and observed state information, the A2C network selects an action $a_{t}\in A$ according to the policy π. These actions consist of resource block selection and transmission power allocation. In this paper, we adopt a simplified scenario in which the actor network could select actions from *m* different resource block groups and three discrete transmission power levels: low, medium, and high. Then the size of the action space is 3m.

#### 4.1.3. Reward Function

In the resource allocation problem, the immediate reward $r_{t}$ is typically designed based on three key criteria: a positive reward is given when the V2V link completes data transmission within the specified time; a negative reward is imposed if the transmission fails; and an additional incentive is provided for achieving successful transmission with lower power consumption, thereby encouraging efficient use of communication resources. The specific reward is calculated in Equation (20).

(20) $$r_{t}=\lambda \cdot R_{V2N}+\left(1-\lambda \right)R_{V2V}-\frac{T_{\mathrm{limit}}-T_{\mathrm{left}}}{T_{\mathrm{limit}}}$$

where $R_{V2N}$ equals the V2N rate, represents the impact of the current action on the V2N communication link; $R_{V2V}$ denotes the V2V transmission success ratio of the current link, corresponding to the impact of the current action on the V2V communication link, λ is a weighting coefficient that balances the importance between V2V and V2N communications, $T_{\mathrm{left}}$ indicates the remaining transmission time for the current task, and $T_{\mathrm{limit}}$ denotes the maximum allowable transmission time.

#### 4.1.4. Actor Network

In the A2C algorithm, the Actor’s policy is described as:

(21) $$\pi (a_{t}|S_{t};\theta_{\pi })$$

where $\theta_{\pi }$ denotes the model parameters of the policy function π, which outputs a probability distribution over actions given an input state. The primary objective of the actor network is to learn an optimal policy as shown in Equation (22).

(22) $$J^{\prime }\left(\theta_{\pi }\right)=\max_{\theta_{\pi }}\left\{J\left(\theta_{\pi }\right)+\beta H\left(\pi \left(\cdot |S_{t};\theta_{\pi }\right)\right)\right\}$$

where

(23) $$J\left(\theta_{\pi }\right)=E_{S_{t},a_{t}}\left[\log\pi \left(a_{t}|S_{t};\theta_{\pi }\right)A\left(S_{t},a_{t}\right)\right]$$

where $\pi (a_{t}|S_{t};\theta_{\pi })$ denotes the probability of taking action $a_{t}$ given state $S_{t}$ under the current policy. $A(S_{t},a_{t})$ is the Advantage function defined in Equation (24):

(24) $$A\left(S_{t},a_{t}\right)=Q\left(S_{t},a_{t};\theta_{Q}\right)-\sum_{a}\pi \left(a|S_{t}\right)Q\left(S_{t},a;\theta_{Q}\right)$$

where *a* represents the set of all possible actions and $a_{t}$ refers to the action sampled from the distribution. $Q(S_{t},a_{t};\theta_{Q})$ represents the state-action value estimated by the Critic network. $\sum_{a}\pi \left(a|S_{t}\right)Q\left(S_{t},a;\theta_{Q}\right)$ represents the value estimation of the current state. If $A\left(S_{t},a_{t}\right)>0$, it indicates that the action performs better than average and its selection probability should be increased; if $A\left(S_{t},a_{t}\right)<0$, it indicates that the action is suboptimal and its selection probability should be decreased.

In Equation (22), β>0 is the entropy regularization coefficient. By incorporating policy entropy regularization, the exploration ability of the policy is preserved, helping to prevent premature convergence to local optima. The entropy is defined in Equation (25):

(25) $$H\left(\pi \left(\cdot |S_{t};\theta_{\pi }\right)\right)=-\sum_{a}\pi \left(a|S_{t};\theta_{\pi }\right)\log\pi \left(a|S_{t};\theta_{\pi }\right)\;$$

Therefore, the gradient of the actor network can be calculated by Equation (26):

(26) $$\nabla_{\theta_{\pi }}J^{\prime }\left(\theta_{\pi }\right)=E_{S_{t},a_{t}}\left[\nabla_{\theta_{\pi }}\log\pi \left(a_{t}|S_{t};\theta_{\pi }\right)A\left(S_{t},a_{t}\right)+\beta \nabla_{\theta_{\pi }}H\left(\pi \left(\cdot |S_{t};\theta_{\pi }\right)\right)\right]\;$$

The final action is selected by choosing the one with the highest probability, as shown in Equation (27).

(27) $$a_{t}=\underset{a}{\mathrm{argmax}}\left(\pi \left(a|S_{t};\theta_{\pi }\right)\right)$$

where *a* represents the set of all possible actions, and $a_{t}$ refers to the action sampled from the distribution. And the Actor network can be updated with Equation (28):

(28) $$\theta_{\pi }\leftarrow \theta_{\pi }+\alpha \nabla_{\theta_{\pi }}J^{\prime }\left(\theta_{\pi }\right)\;$$

where $\theta_{\pi }$ is a trainable parameter of the Actor network.

#### 4.1.5. Critic Network

The goal of the Critic network is to approximate the true action value. we optimize the parameter of the Critic network using the temporal difference (TD) error, which can be expressed as Equation (29).

(29) $$\begin{array}{cc} Loss(\theta_{Q})= & \frac{1}{\left|B\right|}\sum_{(S_{t},a_{t})\in B}{\left(y_{Q}-Q\left(S_{t},a_{t};\theta_{Q}\right)\right)}^{2}\\ y_{Q}= & r_{t}+\gamma Q_{target}\left(S_{t+1},\arg\max_{a\in A}\;\pi_{\mathrm{target}}\left(a|S_{t+1};\theta_{\pi }^{-}\right);\theta_{Q}^{-}\right) \end{array}$$

where $r_{t}$ represents the immediate reward, γ is the discount factor, $\pi_{\mathrm{target}}$ is the target Actor network, $\theta_{\pi }^{-}$ denotes the parameters of the target Actor network, and $\theta_{Q}^{-}$ denotes the parameters of the target Critic network. Therefore, the Critic network can be updated with Equation (30):

(30) $$\theta_{Q}\leftarrow \theta_{Q}+\alpha \nabla_{\theta_{Q}}\mathrm{Loss}\left(\theta_{Q}\right)$$

where $\theta_{Q}$ is a trainable parameter of the Critic network.

Periodically, the parameters of the Actor main networks and Critic main networks are adopted to update their respective target networks to ensure stable learning and convergence every *C* steps with Equation (31):

(31) $$\begin{array}{c} \theta_{\pi }^{-}\leftarrow \tau \theta_{\pi }+\left(1-\tau \theta_{\pi }^{-}\right)\\ \theta_{Q}^{-}\leftarrow \tau \theta_{Q}+\left(1-\tau \theta_{Q}^{-}\right) \end{array}$$

where τ is the learning rate, which is typically set to 0.005 or smaller.

### 4.2. Overall Framework of GAT-A2C Model

**Figure 3:**
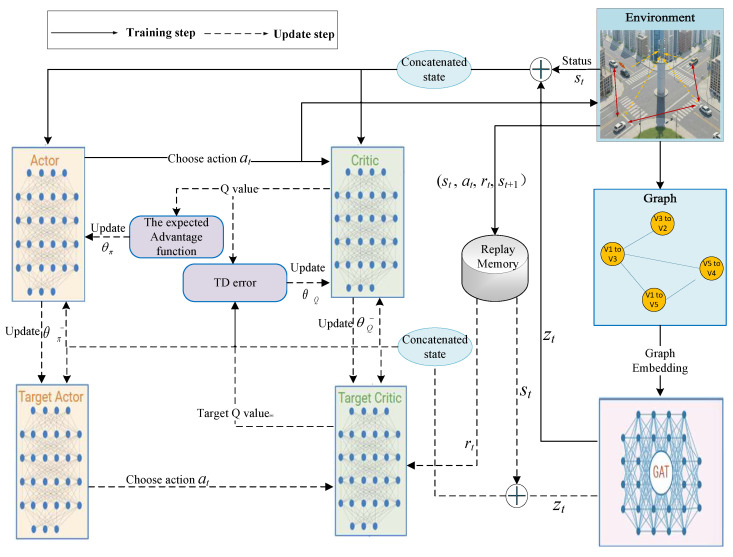
GAT-A2C framework proposed for resource allocation.

Each V2V link operates as an independent agent and executes the decision-making process described in Algorithm 1. The overall framework is as shown in Figure 3. In the framework, each vehicle agent is equipped with the following modules, including the environment module, graph construction module, GAT encoding module, Actor decision-making module, Critic evaluation module, local experience replay, and training module. During the training process, the environment first constructs an adjacency matrix based on the current positions of vehicles. This matrix is then used to form a graph structure representing the V2V links and the interference relationship between them. The resulting graph is then fed into the GAT, where multi-head attention mechanisms are applied to aggregate and enhance the features of neighboring nodes, producing enriched node embeddings.

The proposed system can be regarded as an instance of centralized training and distributed execution. During training, both the GAT and RL components leverage global network information to learn optimal representations and policies. During execution, each node can make decisions based on its own local state and the corresponding GAT embedding, enabling distributed resource allocation without requiring real-time access to global information. This paradigm combines the advantages of global optimization during training with the scalability and practicality of distributed decision-making during deployment.

Within the framework, the GAT module serves as the core feature encoding unit, which not only performs information aggregation but, more critically, equips each RL agent with the ability to perceive the neighbor interference structure. Specifically, the GAT module receives the initialized node features and the dynamically constructed adjacency matrix. The output context-enhanced features serve for action generation in the Actor network and value evaluation in the Critic network.

These embeddings are then concatenated with the original environmental state to form a composite feature vector $S_{t}$, which is input into the actor network. The actor network processes the state vector and outputs an action $a_{t}$ for the corresponding V2V link. Once the action is executed, the environment computes an immediate reward $r_{t}$ according to Equation (20). The environment then updates its state to $S_{t+1}$, and the transition tuple ($S_{t},a_{t},r_{t},S_{t+1}$) is stored in the replay memory.

During the update phase, mini-batches *B* are sampled from the replay memory and fed into the respective networks to update the actor, critic, and GAT networks.
**Algorithm 1** ResourceAllocationAlgorithm().**Require:** Local state $S_{t}$, GAT model, Actor–Critic model, Maximum iteration counter      
max_iter, Current iteration counter iter←0, Convergence threshold ϵ, converged←      *False*
**Ensure:** GAT Network parameter $\theta_{GAT}$, Actor Network parameter $\theta_{\pi }$, Critic Network      parameter $\theta_{Q}$, Target Network parameter $\theta_{\pi }^{-}$, $\theta_{Q}^{-}$, Replay Memory Buffer *D*;
 1:  **while**
iter < max_iter and converged = False **do** 2:      Initialize Environment, get local State $s_{t}$, 3:      //Step 1: Build Adjacency matrix(dynamic Graph)
 4:      Build dynamic Graph G=(N,E) 5:      //Step 2: GAT aggregates neighbor information
 6:      $z_{t}=GAT\left(G\right)$
 7:      //Step 3: State concatenation
 8:      $S_{t}=s_{t}\left|\right|z_{t}$
 9:      //Step 4: action selection (Actor outputs resource blocks & power)
10:      $\pi (a_{t}|S_{t};\theta_{\pi })$
11:      //Step 5: Critic scores the current state
12:      $Q(S_{t},a_{t};\theta_{Q})$
13:      //Step 6: The agent executes actions on the environment, and the environment
           provides feedback to the agent.
14:      Get $r_{t}$
15:      //Step 7: Store experiences
16:      Store ($s_{t}$,*a*,$r_{t}$,$s_{t+1}$) into the Replay Memory Buffer *D*
17:      //Step 8: Update Network
18:      **if** reached the update period **then**
19:         //Batch sample B instances
20:         Batch sample B instances from Replay Memory Buffer D ← Batch
21:         //Calculate the TD(Temporal Difference) target value
22:         $y_{t}=r_{t}+\gamma Q\left(s_{t}^{\prime },a_{t}^{\prime };\theta_{Q}^{-}\right)$
23:         //Update the Critic network by minimizing the TD error
24:         $\theta_{Q}\leftarrow \theta_{Q}-\alpha \nabla Loss_{Q}$
25:         //Calculate the Advantage
26:         $A\left(S_{t},a_{t}\right)=Q\left(S_{t},a_{t}\right)-V\left(S_{t}\right)$
27:         //Update the Actor network by maximizing the expected advantage function
28:         $\theta_{\pi }\leftarrow \theta_{\pi }+\alpha \nabla_{\theta_{\pi }}J^{\prime }\left(\theta_{\pi }\right)$
29:         //Soft update the target network parameters
30:         $\theta_{Q}^{-}\leftarrow \tau \theta_{Q}+\left(1-\tau \right)\theta_{Q}^{-}$
31:         $\theta_{\pi }^{-}\leftarrow \tau \theta_{\pi }+\left(1-\tau \right)\theta_{\pi }^{-}$
32:      **end if**
33:      iter←iter+1
34:  **end while**


The A2C algorithm consists of two main components: the actor, which represents the policy function and determines the agent’s actions, and the critic, which serves as the value function to evaluate the value of those actions. Based on the rewards returned by the environment, the system adjusts the actor if its action was suboptimal, and corrects the critic if its evaluation was inaccurate. Through continuous updates and iterations, the model gradually learns to output more optimal actions by the approach to maximize the expected return. From the perspective of V2V links, this means that the quality and reliability of V2V communications will progressively improve.

### 4.3. Time Complexity Analysis and Limitation Discussion

#### 4.3.1. Time Complexity of GAT-A2C Model

The proposed GAT-A2C model comprises two main components: the GAT module and the A2C algorithm module. In this section, we consider a communication system with *n* vehicles and analyze the time complexity of each component separately.

In the GAT module, graph construction and forward-propagation are two key steps that consume time. In the graph construction process, the total number of nodes is m=3n, as each vehicle is associated with three communication links. Computing the distance matrix requires $O(n^{2})$ operations. Neighbor determination involves sorting the distance for each of the *n* nodes, resulting in a complexity of $O\left(n\cdot n\log n\right)=O\left(n^{2}\log n\right)$. Expanding the adjacency matrix requires $O\left(m^{2}\right)=O\left(n^{2}\right)$ element assignments. In the forward-propagation process, the time complexity of feature transformation is O(m·F·H)=O(nFH), where *F* denotes the number of hidden layers and *H* the number of attention heads. The computation of the attention score dominates the complexity, requiring $O(n^{2}H)$ operations, which include feature concatenation, linear transformation, LeakyReLU activation, and softmax normalization. Feature aggregation has a computational complexity of O(m·E·H), which simplifies to $O(n^{2}H)$ in dense scenarios. Here, E≃n represents the average number of neighbors that can cause significant interference. Overall, the time complexity of the GAT module is dominated by $O(n^{2}H)$.

The A2C module comprises two fully connected neural networks: the actor {Inputlayer(state)→Hiddenlayer1→Hiddenlayer2→Outputlayer(action)} and the critic {Inputlayer(state)→Hiddenlayer1→Hiddenlayer2→Outputlayer(value)}. In the Actor network, the four layers have dimensions 102, 500, 250, and 60, respectively, resulting in a total complexity of approximately $1.95\times 10^{5}$ operations per forward pass. In the Critic network, the four layers have dimensions 102, 500, 250, and 1, with each forward pass requiring approximately $1.76\times 10^{5}$ operations. For Batch training with a batch size of B=2000, and assuming the backward pass has a similar computational cost to the forward pass, the total number of operations is approximately $7.38\times 10^{5}\times B$.

Overall, for *n* vehicles with three decision points each, the total computational complexity of each iteration is dominated by the GAT forward-propagation term, $O\left(3n\cdot n^{2}H\right)=O\left(n^{3}H\right)$, and the A2C network inference term, O(3n×B). For large *n*, the GAT component is the primary computational bottleneck.

#### 4.3.2. Limitation Discussion

Based on the above analysis of computational complexity, the GAT-A2C model appears to be potentially deployable with current technological capabilities. The overall complexity of the proposed approach is primarily determined by the cube of the number of vehicles $\left(n^{3}\right)$ and the number of attention heads *H*. Consequently, this model is most suitable for scenarios involving a relatively small number of vehicles. As the number of vehicles increases significantly, the execution time of the entire model grows substantially, which may hinder real-time computation.

A potential solution is a pretrain-and-deploy strategy. In this approach, the model is first trained in scenarios with a relatively small number of vehicles and then directly applied to environments with higher vehicle density. This method improves the model’s applicability in high-density scenarios at the cost of a modest reduction in accuracy.

## 5. Experiment

We conduct the simulation experiments to evaluate the effectiveness of the proposed GAT-A2C strategy for resource allocation in vehicular networks. First, we monitored the training details of the GAT separately, including the training loss, V2N rate, and V2V success ratio with step, to analyze the training process. Then, we compare the performance of the proposed model with different densities. Thirdly, we compare the proposed model with the other three methods by comparing the proposed approach against three baseline methods: random resource allocation, a standard DQN model, and a GNN-DDQN model. The experiment results show the advantages of GAT-A2C in optimizing V2N rate and V2V success ratio. Fourthly, we conduct the ablation experiments to observe the effect of the GAT model in the proposed model. Finally, more ablation experiments are conducted to explore the impact of attention head number and attention layer number in the proposed model. In this section, we present the experimental setup and experimental results in detail.

### 5.1. Experimental Settings

We conduct the simulation experiments using Python 3.8 and PyTorch 2.0, adopting settings similar to those in [12,20]. Simulation is a widely used tool in the research of vehicular networks due to its high flexibility and low cost [26,27,28], making it particularly suitable for the study in this paper. Furthermore, prior studies, such as those on dedicated short-range communications (DSRC) communication systems [29], have demonstrated that simulation-based assessments can achieve results comparable to those obtained from emulation-based methods. This evidence supports the reliability of simulation approaches, confirming their effectiveness as a viable method for our research. In the simulation experiments, we established a Manhattan grid traffic road scenario with a radio communication frequency band of 2 GHz, following the scenario settings described in 3GPP TR 36.885, encompassing both line-of-sight (LOS) and non-line-of-sight (NLOS) channel conditions [30]. The vehicles are placed according to a Poisson distribution. Each vehicle’s speed, acceleration, and other related information were assigned via a Gaussian distribution.

We employed a GAT model with a depth of two, using eight attention heads in the first layer and incorporating edge weights to reflect varying degrees of interaction between communication links. The feature dimension extracted by GAT was set to 16. The input feature dimension for each node in the graph network was 60, with a self-attention mechanism used for feature aggregation. For the A2C model, the state input dimension was 102. Both the actor and critic networks adopted a three-layer neural network architecture with 500, 250, and 120 neurons per layer, respectively. The actor network outputs a probability distribution over 60 actions, while the critic network estimates the value of the state-action pair. A Rectified Linear Unit (ReLU) is used as the activation function between layers. The learning rate was set to decrease gradually during training. For the GAT network, the initial learning rate was 0.01, with a decay factor of 0.96, and the final learning rate was $10^{-4}$. For the A2C network, the initial learning rate was 0.005, and the final learning rate was $10^{-4}$. Other detailed parameter settings are provided in Table 1.

### 5.2. Experiment Results

#### 5.2.1. Training Loss and Effectiveness of the GAT-A2C Model

**Figure 4:**
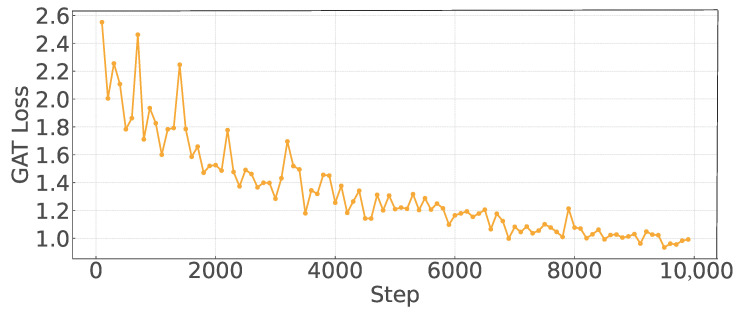
GAT training loss vs. Step.

Figure 4 shows the loss performance of the GAT-A2C model in the training process when the vehicle number is 20. To optimize computational efficiency, the GAT-A2C model is updated every 100 iterations. We observed that the loss converged quickly, although the environment was reset every 2000 iterations and the relationship of the graph was rebuilt, which caused the loss value to fluctuate significantly in the early stage. However, as the training progressed, it can be seen that the amplitude of the fluctuation is constantly decreasing, which shows that the GAT-A2C model can adapt to the environment.

**Figure 5:**
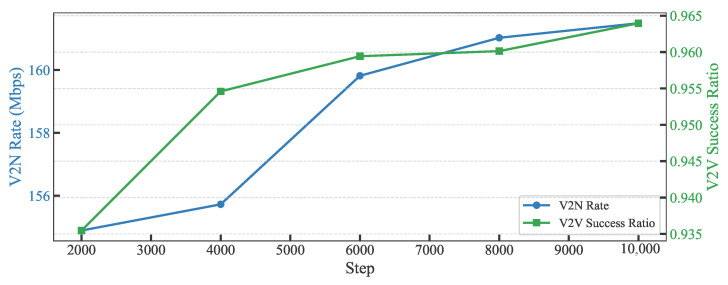
V2N rate and V2V success ratio vs. Step.

Figure 5 presents the effectiveness of the GAT-A2C model in optimizing the performance of V2V and V2N communications when the vehicle number is 20. The results indicate that the V2V success ratio increases steadily from 0.935 to 0.965, a rise of approximately 3%, demonstrating that reinforcement learning—through strategies like the RL Agent’s policy optimization and the GAT’s spatial relationship learning—significantly enhances the reliability of V2V communication. Concurrently, the V2N rate improves from 155 to approximately 160.5, reflecting enhanced efficiency in infrastructure interaction. The contrasting trends, with the V2V success ratio showing consistent growth and the V2N rate stabilizing after an initial increase, confirm that reinforcement learning iteratively refines resource allocation and interference management, providing experimental evidence for the system’s performance improvement.

#### 5.2.2. Performance Analysis of GAT-A2C at Different Densities

**Figure 6:**
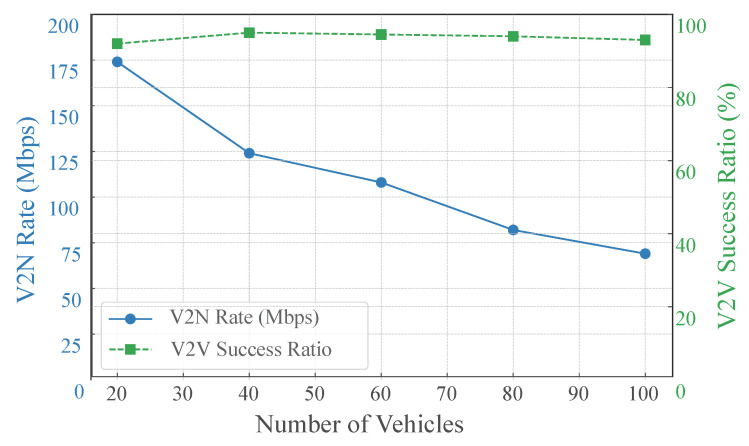
V2N Rate and V2V Success Ratio vs. Vehicle Number.

We evaluated the performance of the proposed GAT-A2C strategy on V2N Rate and V2V Success Ratio as the number of vehicles increased from 20 to 100 vehicles. As shown in Figure 6, the experimental results indicate that as the number of vehicles grows, the number of links in the system surges, intensifying resource block competition and interference. Consequently, the V2N rate exhibits a clear downward trend, dropping from approximately 170 Mbps to around 70 Mbps. Remarkably, despite the vehicle density doubling, the V2V success ratio consistently remains above 90% with minimal fluctuations, demonstrating strong robustness. This can be attributed to the introduction of the GAT, which enables agents to obtain contextually richer state representations, combined with the Actor–Critic strategy’s long-term value assessment mechanism for resource selection. This ensures efficient scheduling even under high vehicle density. Thus, despite some V2N performance trade-offs, the proposed strategy showcases excellent scalability and scheduling robustness in ensuring core V2V service guarantees.

**Figure 7:**
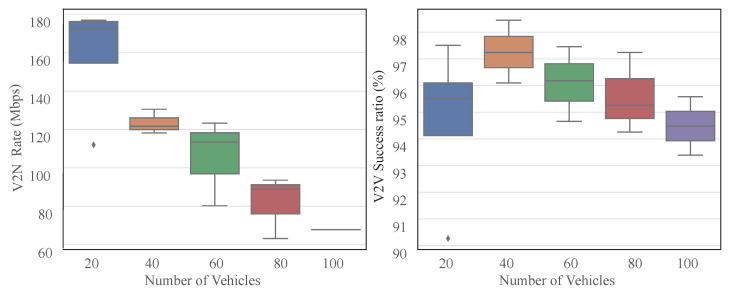
V2N Rate Distribution and V2V Success Ratio Distribution vs. Vehicle Number.

The boxplots in Figure 7 reveal distinct distribution patterns: V2N rates exhibit decreasing mean values with increasing vehicle density, reflecting intensified interference impacts on resource scheduling. Conversely, V2V success ratios demonstrate remarkable robustness, maintaining medians above 0.93 with minimal fluctuations across densities. Optimal V2V performance occurs at 40 vehicles, showing both peak success ratio and minimal variance. Notably, this outperforms sparse scenarios (e.g., 20 vehicles) since the graph attention mechanism requires sufficient node connectivity and the A2C framework benefits from diverse interaction experiences for optimal policy learning. Occasional low values during initialization confirm the system’s recovery capability, collectively validating the approach’s robustness in high-density environments, particularly for core V2V link maintenance.

#### 5.2.3. Performance Analysis Compared with Other Methods

Three baseline methods are employed for comparison. The first is a random resource allocation approach, where the agent arbitrarily assigns channels and power levels, establishing a lower performance benchmark. The second, presented in [12], utilizes a standard DQN model for resource allocation. The third, described in [20], integrates GNN with a DDQN to enable each agent to acquire more information from local observations.

**Figure 8:**
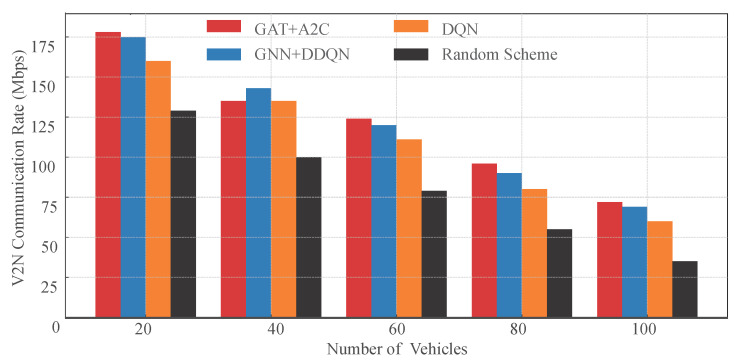
V2N Rate compared with the other methods.

We compared the V2N communication performance of these four resource allocation strategies under varying vehicle density conditions. As shown in Figure 8, the experimental results demonstrate the significant advantage of the proposed GAT-A2C strategy in terms of V2N rate. As the number of vehicles increases from 20 to 100, the number of interfering links in the system multiplies, intensifying resource block competition, and the V2N rate of all strategies shows a declining trend. However, at each vehicle density, GAT-A2C consistently maintains the highest V2N rate, with a more pronounced lead in high-density scenarios (80 and 100 vehicles). Compared to the traditional DQN method, GAT-A2C achieves over 20% higher V2N rate in the 100-vehicle scenario, and nearly doubles compared to the random strategy. This phenomenon is attributed to the critical role of the Graph Attention Network in state modeling, enabling the strategy to accurately identify high-interference links and make avoidance-driven resource selections. Additionally, the policy gradient optimization mechanism of the A2C architecture enhances the flexibility of resource allocation, avoiding extreme action decisions and improving resource utilization efficiency.

**Figure 9:**
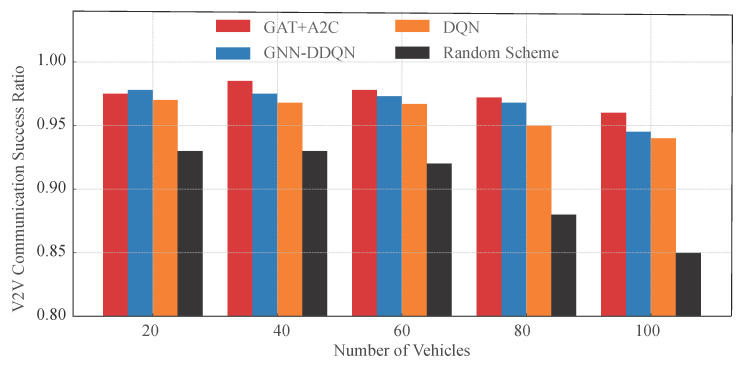
V2V Success Ratio compared with the other methods.

Figure 9 illustrates the V2V link transmission success ratios of different strategies under various vehicle densities, further confirming the effectiveness of the proposed GAT-A2C strategy in ensuring link service guarantees. Overall, GAT-A2C and GNN-DDQN consistently rank highest in V2V success ratio, particularly exhibiting the most stable performance in medium-to-high-density scenarios (40–80 vehicles), with success ratios maintained above 96%. In contrast, the standard DQN method shows a significant decline in scenarios with 80 and 100 vehicles, while the random strategy drops below 90%, highlighting the limitations of traditional methods in high-interference environments. Notably, although both GNN-DDQN and the proposed method utilize graph embedding mechanisms, the A2C architecture demonstrates superior policy learning capabilities, dynamically adapting to changes in link states and task urgency, thus offering greater stability under high-density conditions. Combined with Figure 8, it is evident that the proposed strategy not only ensures high V2V success ratios but also balances V2N rate, showcasing excellent scheduling coordination and overall system service capability. These results validate the feasibility of deeply integrating graph neural network structures with policy-based reinforcement learning methods, effectively addressing the core challenges of multi-link resource allocation in V2X communication.

#### 5.2.4. The Effect of GAT in the Proposed Model

**Figure 10:**
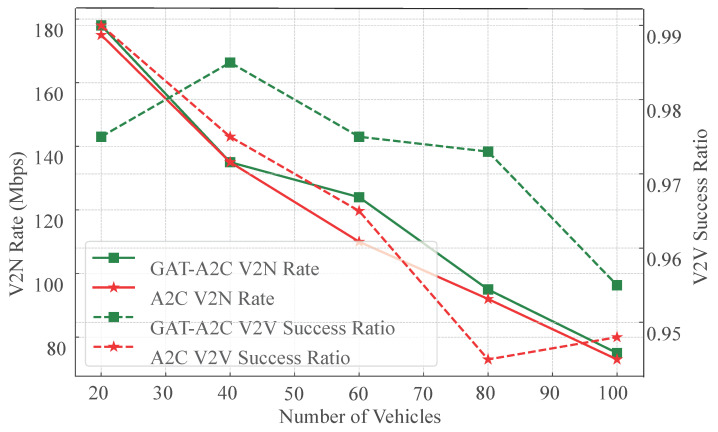
V2N Rate and V2V success ratio under GAT-A2C method (*w*/ vs. *w*/*o* GAT).

Figure 10 presents a comparative analysis of the proposed GAT-A2C strategy versus a pure A2C strategy without graph structure embedding, evaluating V2N rate and V2V success ratio across varying vehicle numbers. The experimental results clearly demonstrate that while both strategies perform similarly in low-density scenarios (20–40 vehicles), GAT-A2C exhibits significantly greater robustness and scalability in medium-to-high density environments.

For the V2N rate, both strategies show a declining trend as vehicle density increases. However, GAT-A2C consistently maintains higher transmission rates, particularly in environments with 80 and 100 vehicles, where its rate surpasses A2C by 10–15 Mbps. This indicates that the incorporation of GAT enables the strategy to more accurately perceive interference during resource block selection, mitigating conflict escalation in high-density scenarios.

Regarding the V2V success ratio, GAT-A2C not only achieves superior overall performance (consistently above 95%) but also exhibits a more gradual decline. In contrast, the A2C strategy experiences a rapid drop in success ratio when the vehicle number exceeds 60, highlighting its significant limitations in multi-link resource coordination. Combined with earlier power distribution experiments, it is evident that GAT-A2C proactively increases the proportion of medium-to-low power selections in dense vehicle scenarios, whereas A2C exhibits rigid behavior and uniform distribution, failing to adapt flexibly to environmental changes.

In summary, this figure strongly validates the critical role of graph structure awareness in reinforcement learning strategies for large-scale vehicular networks. GAT not only enhances state representation capabilities but also provides a robust foundation for modeling link dependencies, enabling the strategy to maintain strong service guarantees and optimization flexibility in high-density, high-interference, and dynamic topology scenarios. The scheduling stability and high success ratio demonstrated by the GAT-A2C model in complex environments underscore its core advantage as a deployable scheduling solution for vehicular networks.

**Figure 11:**
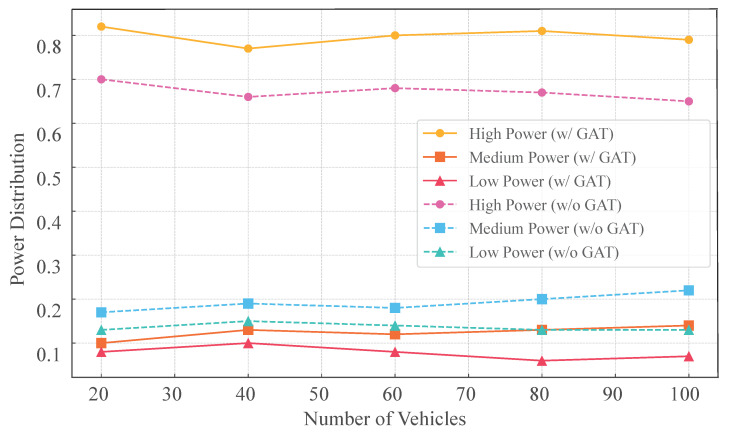
Power selection distribution under GAT-A2C method (*w*/ vs. *w*/*o* GAT).

Figure 11 illustrates how the proposed GAT-A2C strategy adjusts the distribution of three transmission power levels (high, medium, low) across varying vehicle densities (20 to 100 vehicles). It is evident that high-power selection consistently prevails (75–82%) across all density levels, maximizing the transmission range of data packets. This preference enhances the strategy’s ability to ensure robust data delivery, particularly by extending the reach of transmissions. The incorporation of contextual link interference information from the GAT model further reinforces this tendency. Compared to the A2C strategy without GAT, the incorporation of the GAT module markedly elevates the predilection for high-power utilization, capitalizing on structural information embedded within state modeling. This figure confirms, from a behavioral strategy perspective, the enhanced control and adaptability provided by GAT, underscoring the superior effectiveness and robustness of the GAT-A2C strategy in high-density V2X communication systems.

#### 5.2.5. The Effect of Attention Head Number and Attention Layer Number in the Proposed Model

**Figure 12:**
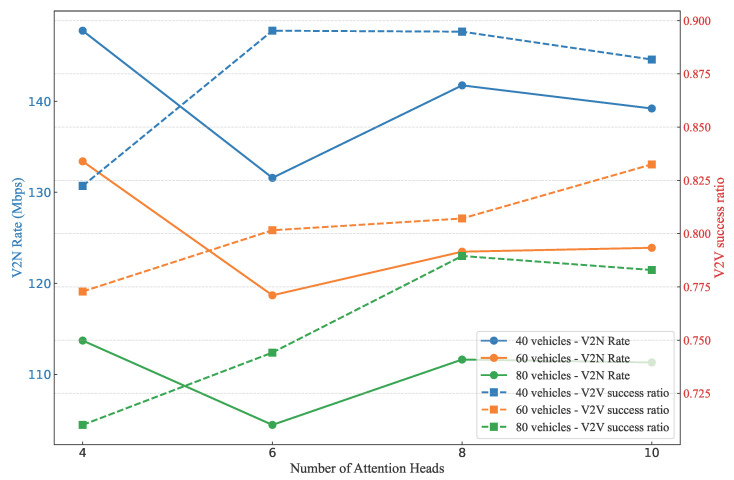
Comparisons of different attention head numbers under the GAT-A2C method.

We conduct ablation experiments to explore the impact of attention head number and attention layer number in the proposed model. First, we analyzed the effects of varying attention head numbers (four, six, eight, and ten) across a range of vehicle counts (20 to 100). Figure 12 presents the results. As shown in Figure 12, The results show that V2N rates generally increase with more attention heads up to a certain point, peaking around eight heads for all vehicle densities—reaching approximately 130–140 Mbps—before potentially declining or stabilizing at ten heads, suggesting that excessive heads may introduce overfitting or computational overhead without proportional gains. Similarly, V2V success ratios improve steadily from four to eight heads, achieving higher values (around 0.85–0.90) in denser scenarios, but show diminishing returns or slight drops at ten heads, indicating that too many heads could exacerbate interference modeling complexities in dynamic environments. This trade-off highlights that eight attention heads strike an optimal balance between capturing intricate spatial relationships and maintaining efficiency, leading to our selection of eight heads in the final experiment for robust performance across vehicle densities.

**Figure 13:**
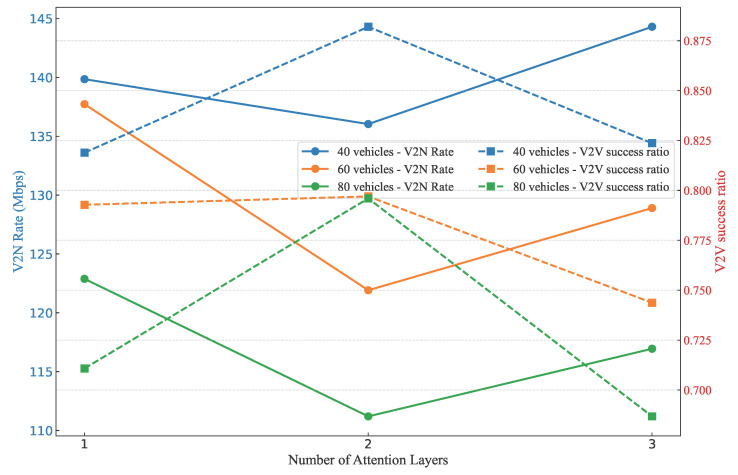
Comparisons of different attention layer numbers under the GAT-A2C method.

The ablation experiment on the number of attention layers, as illustrated in the Figure 13, evaluates the performance of V2N rates and V2V success ratios under configurations of one, two, and three layers across vehicle densities of 40, 60, and 80. V2N rates, ranging from 110 to 145 Mbps, exhibit a pattern where values are highest at one layer, decline to the lowest at two layers, and subsequently rise at three layers for all vehicle counts. In contrast, V2V success ratios, spanning 0.700 to 0.875, are lowest at one layer, reach their peak at two layers, and then decrease at three layers across the tested densities. This analysis underscores the trade-off between the two metrics, leading to the selection of two attention layers in our experiment to prioritize reliable V2V communication, while maintaining V2N rates within an acceptable range.

## 6. Conclusions

This paper addresses the significant challenge of dynamic communication resource allocation in vehicular networks by proposing a novel framework that integrates Graph Attention Networks with Advantage Actor–Critic (GAT-A2C) Deep Reinforcement Learning. This framework innovatively combines GAT with the Advantage Actor–Critic RL paradigm, effectively tackling the resource allocation challenges in dynamic vehicular network environments. Additionally, the method dynamically updates the adjacency matrix based on real-time vehicle mobility and channel conditions, ensuring that the GAT module accurately reflects the current network topology. This is crucial for maintaining communication performance in highly dynamic vehicular environments.

The GAT-A2C framework demonstrates robust training performance, with its GAT loss function converging rapidly after initial fluctuations, showcasing strong learning stability. Comprehensive experimental evaluations confirm the superiority of the proposed method across various vehicle densities. The GAT-A2C framework consistently achieves a V2V communication success ratio above 95%, with minimal degradation as vehicle numbers increase from 20 to 100, highlighting exceptional link service assurance. While the V2N rate declines with rising vehicle density, GAT-A2C outperforms baseline methods like DQN and Actor–Critic without graph structures, achieving over 20% higher rates than traditional DQN in a 100-vehicle scenario. The strategy consistently favors high-power transmission (75–82%) across density levels, enhancing data delivery range and robustness. By integrating contextual link interference information, the GAT module further strengthens this preference and enhances adaptability, ensuring superior performance compared to other methods.

This study assumes that all vehicles are equipped with communication devices. In practice, however, achieving a 100% penetration rate of connected vehicles is unlikely in the near future. Therefore, future work will extend this research to include scenarios with non-connected vehicles, in order to better reflect real-world traffic conditions and evaluate the robustness of our approach in mixed traffic environments. Additionally, we plan to integrate our current GAT-A2C framework with multi-agent reinforcement learning (MARL) techniques, such as mean-field MARL or cascaded MADDPG variants, to further enhance its capabilities and enable more meaningful contributions in vehicular scenarios. 

## Figures and Tables

**Table 1:** Parameter settings.

Description	Specification	Description	Specification
Scenario	Intersection	Dis. threshold for neighbor vehicles	150 m
Number of lanes	(2+2)×4	Weight coefficients [$\lambda_{r}$,$\lambda_{p}$]	[0.3, 1.0]
Vehicle speed	36∼54 km/h	GAT input feature dimension	60
Packet size	1500 bytes	GAT output embedding dimension	20
Avg. V2V pkt generation rate	20 Hz	Number of GAT attention heads	8
Carrier frequency	2 GHz	GAT dropout rate	0.6
Total number of RBs	20	State input dimension to actor–critic	102 (82 base + 20 GAT)
Antenna gain of veh. & BS	3 dBi & 8 dBi	Replay memory capacity	1 million
Antenna height of veh. & BS	1.5 m & 25 m	Replay batch size	2000
Noise figure of veh. & BS	9 dB & 5 dB	Learning rate	0.01 (min $10^{-4}$, decay 0.96)
Noise power	−114 dBm	Discount factor λ	0.5
Maximum delay for V2V link	100 ms	Soft target update rate τ	0.01
Transmission power levels	[23, 10, 5] dBm	Training steps	10000

## Data Availability

Dataset available on request from the authors.

## References

[1] Lu N., Cheng N., Zhang N., Shen X., Mark J. (2014). Connected Vehicles: Solutions and Challenges. IEEE Internet Things J..

[2] Wen X., Chen J., Hu Z., Lu Z. (2020). A p-Opportunistic Channel Access Scheme for Interference Mitigation Between V2V and V2I Communications. IEEE Internet Things J..

[3] Fang Y. (2015). Connected Vehicles Make Transportation Faster, Safer, Smarter, and Greener!. IEEE Trans. Veh. Technol..

[4] Sehla K., Nguyen T.M.T., Pujolle G., Velloso P.B. (2022). Resource Allocation Modes in C-V2X: From LTE-V2X to 5G-V2X. IEEE Internet Things J..

[5] Gonzalez-Martín M., Sepulcre M., Molina-Masegosa R., Gozalvez J. (2019). Analytical Models of the Performance of C-V2X Mode 4 Vehicular Communications. IEEE Trans. Veh. Technol..

[6] Yuan Y., Zheng G., Wong K.K., Letaief K.B. (2021). Meta-Reinforcement Learning Based Resource Allocation for Dynamic V2X Communications. IEEE Trans. Veh. Technol..

[7] Ji B., Dong B., Li D., Wang Y., Yang L., Tsimenidis C., Menon V.G. (2023). Optimization of resource allocation for V2X security communication based on multi-agent reinforcement learning. IEEE Trans. Veh. Technol..

[8] Yang H., Xie X., Kadoch M. (2019). Intelligent Resource Management Based on Reinforcement Learning for Ultra-Reliable and Low-Latency IoV Communication Networks. IEEE Trans. Veh. Technol..

[9] Gyawali S., Qian Y., Hu R.Q. Resource Allocation in Vehicular Communications Using Graph and Deep Reinforcement Learning. Proceedings of the 2019 IEEE Global Communications Conference (GLOBECOM).

[10] Li R., Zhao Z., Chen X., Palicot J., Zhang H. (2014). TACT: A Transfer Actor-Critic Learning Framework for Energy Saving in Cellular Radio Access Networks. IEEE Trans. Wirel. Commun..

[11] Veličković P., Cucurull G., Casanova A., Romero A., Lio P., Bengio Y. (2017). Graph Attention Networks. arXiv.

[12] Ye H., Li G.Y., Juang B.H.F. (2019). Deep Reinforcement Learning Based Resource Allocation for V2V Communications. IEEE Trans. Veh. Technol..

[13] Zhao D., Qin H., Song B., Zhang Y., Du X., Guizani M. (2020). A Reinforcement Learning Method for Joint Mode Selection and Power Adaptation in the V2V Communication Network in 5G. IEEE Trans. Cogn. Commun. Netw..

[14] Nguyen K.K., Duong T.Q., Vien N.A., Le-Khac N.A., Nguyen L.D. (2019). Distributed Deep Deterministic Policy Gradient for Power Allocation Control in D2D-Based V2V Communications. IEEE Access.

[15] Miao J., Chai X., Song X., Song T. A DDQN-based Energy-Efficient Resource Allocation Scheme for Low-Latency V2V communication. Proceedings of the 2022 IEEE 5th International Electrical and Energy Conference (CIEEC).

[16] Gao A., Wang Q., Wang Y., Du C., Hu Y., Liang W., Ng S.X. (2024). Attention Enhanced Multi-Agent Reinforcement Learning for Cooperative Spectrum Sensing in Cognitive Radio Networks. IEEE Trans. Veh. Technol..

[17] Chen T., Zhang X., You M., Zheng G., Lambotharan S. (2022). A GNN-Based Supervised Learning Framework for Resource Allocation in Wireless IoT Networks. IEEE Internet Things J..

[18] Guo J., Yang C. (2022). Learning Power Allocation for Multi-Cell-Multi-User Systems With Heterogeneous Graph Neural Networks. IEEE Trans. Wirel. Commun..

[19] He Z., Wang L., Ye H., Li G.Y., Juang B.H.F. Resource Allocation based on Graph Neural Networks in Vehicular Communications. Proceedings of the GLOBECOM 2020—2020 IEEE Global Communications Conference.

[20] Ji M., Wu Q., Fan P., Cheng N., Chen W., Wang J., Letaief K.B. (2025). Graph Neural Networks and Deep Reinforcement Learning-Based Resource Allocation for V2X Communications. IEEE Internet Things J..

[21] Yuan C., Zhao H., Yan W., Hou L. Resource Allocation with Multi-Level QoS for V2X Based on GNN and RL. Proceedings of the 2023 International Conference on Information Processing and Network Provisioning (ICIPNP).

[22] Zhang M., Chen Y. (2018). Link Prediction Based on Graph Neural Networks. arXiv.

[23] Kipf T.N., Welling M. (2016). Semi-supervised classification with graph convolutional networks. arXiv.

[24] Zhang M., Cui Z., Neumann M., Chen Y. An End-to-End Deep Learning Architecture for Graph Classification. Proceedings of the AAAI Conference on Artificial Intelligence.

[25] Ioffe S., Szegedy C. Batch normalization: Accelerating deep network training by reducing internal covariate shift. Proceedings of the 32nd International Conference on International Conference on Machine Learning.

[26] Zhuofei W., Bartoletti S., Martinez V., Bazzi A. (2023). Adaptive repetition strategies in IEEE 802.11 bd V2X networks. IEEE Trans. Veh. Technol..

[27] Todisco V., Bartoletti S., Campolo C., Molinaro A., Berthet A.O., Bazzi A. (2021). Performance analysis of sidelink 5G-V2X mode 2 through an open-source simulator. IEEE Access.

[28] Li Z., Wang Y., Zhao J. (2022). Reliability Evaluation of IEEE 802.11p Broadcast Ad Hoc Networks on the Highway. IEEE Trans. Veh. Technol..

[29] Bansal G., Kenney J.B., Weinfield A. Cross-Validation of DSRC Radio Testbed and NS-2 Simulation Platform for Vehicular Safety Communications. Proceedings of the 2011 IEEE Vehicular Technology Conference (VTC Fall).

[30] 3GPP (2017). 3rd Generation Partnership Project; Technical Specification Group Radio Access Network; Evolved Universal Terrestrial Radio Access (E-UTRA); Further advancements for E-UTRA physical layer aspects (Release 9). Technical Report (TR) 36.814. https://www.3gpp.org/ftp//Specs/archive/36_series/36.814/36814-920.zip.

